# Clinical Lectures on Certain Acute Diseases

**Published:** 1860-09

**Authors:** 


ART. III.—*Clinical Lectures on Certain Acute Diseases.* By ROBERT BENTLEY TODD, M.D., F.R.S., etc. etc. 8vo., pp. 308. Philadelphia: Blanchard & Lea, 1860.

THERE is at the present time in Great Britain a formidable array of names enlisted against the so-called antiphlogistic treatment of disease; and however much the doctrine may come in conflict with our previous notions, it must be conceded that with such advocates as Sir John Forbes, John Hughes Bennett, and Robert Bentley Todd, there is at least a very respectable weight of authority against the depletory system of practice, even in acute inflammatory diseases. It may be questionable, however, notwithstanding the justly proud eminence which these names occupy in the history of our literature, whether the views so enthusiastically espoused by the advocates of the new theory are based upon an untenable hypothesis respecting the nature of inflammation, or upon legitimately and accurately observed clinical facts. Medicine as a science always influences, to a greater or less degree, medicine as an art; our practice is apt to partake of our theory; and in the Clinical Lectures of Dr. Todd, we have a bold *practical* exposition of the school to which he belongs, and the theory which he so long and so ably advocated. This will appear evident from the following propositions which are briefly summed up in the preface to his Lectures:—

"1. That the notion so long prevalent in the schools, that acute disease can be prevented or cured by means which depress and reduce vital and nervous power, is altogether fallacious.

"2. That acute disease is not curable by the direct influence of any form of drug, or any known remedial agent, excepting when it is capable of acting as an antidote, or of neutralizing a poison, on the presence of which in the system the disease may depend, (*materies morbi.*)

"3. That disease is cured by natural processes, to promote which, in their full vigor, vital power must be upheld. Remedies, whether in the shape of drugs which exercise a special physiological influence on the system, or in whatever form, are useful only so far as they may excite, assist, or promote these natural curative processes.

"4. That it should be the aim of the physician (after he has sedulously studied the clinical history of disease and made himself master of its diagnosis) to inquire minutely into the intimate nature of the curative processes—their physiology, so to speak—to discover the best means of assisting them, to search for antidotes to morbid poisons, and to ascertain the best and most convenient methods of upholding vital power."

The practice of Dr. Todd, it will be at once perceived, is based upon the hypothesis of Sir John Forbes, that all diseases are but modifications of physiological action, that they are essentially vital, and being but manifestations of modified vital action, the same *a priori* grounds are assumed by our author throughout, that nature alone has the power of curing diseases, and that art can assist only so far as she can aid nature in her salutary and conservative reactions. Until quite recently he would have been regarded as a bold speculator, and a worse practitioner, who would have maintained this view in reference to the treatment of acute inflammatory affections; and yet if we assent to the premises assumed by the advocates of the new pathology, we cannot consistently, at least so far as theory is concerned, refuse to accept the practical deductions. It is proper, therefore, that we should carefully scrutinize the premises of the advocates of a practice so largely at variance with the concurrent testimony of the great body of the profession.

Some years since we remember to have read an interesting monograph on the subject of inflammation, by Professor Watters, of St. Louis, in which the views of Dr. Todd, Bennett, and others were clearly foreshadowed. It was assumed in the essay to which we refer that "*disintegration is the vital motor*," and that we must look to destruction of tissue for the primary condition of increased capillary circulation, or "capillary power," as it is sometimes termed. The same view was subsequently presented by Dr. Hinton, in the *British and Foreign Medico-Chirurgical Review* for July, 1858. He there assumes, under a somewhat varied phraseology, the same general position, viz., that "increased action of inflammation is the *effect* of increased decay;" and he reasons from this, that the symptoms of increased activity in the inflammatory process can never go beyond their *cause*, can never exceed the defect of vitality of which they are at once the effect and the sign, and that the reactive process in inflammation is in every case a *salutary*, that is, a saving or restorative process.

Dr. Bennett, in his Clinical Lectures on Inflammation, presents, substantially, the same view. He regards the throbbing of the vessels witnessed during inflammation as a *result*, not a cause of the morbid action. According to his view the blood is not forced in by a *vis a tergo*, but is drawn in by a *vis a fronte*. With Hinton he contends that the hyperæmia is salutary, the increased vascularity in the inflamed part being a wise provision of nature to promote cell growth in the part. Hence, says Bennett, "as cell growth occurs during inflammation, it is absolutely imperative that the part in which these nutritive changes go on should receive more blood to enable it to accomplish them." Blood-letting, therefore, by a most logical process of reasoning, becomes, in his estimation, an improper agent in the treatment of inflammation; for the reason that it interferes with the nutritive acts necessary to accomplish a cure. And Dr. Todd assents to this doctrine by adding: "You must feed inflammations as you would other active vital processes."

Now with all deference to the eminent authority of Forbes, Bennett, Todd and others, we cannot but regard the doctrine of *pathological* nature being conservative in her reactions as liable to many exceptions, not the least striking of which is that of acute inflammation; nor can we for a moment think, notwithstanding the high authority in its support, that the practice of attempting to force blood by the free use of artificial stimulants, into inflamed tissues in the acute phlegmasia, will ever receive the sanction of any considerable number of practitioners either in Great Britain or America.

And just here the question may be naturally propounded,—Why give stimulants? If, as is assumed by the advocates of the new theory, the increased action of the vessels is but an expression of a want in the cell structure of the part, and that the inflammatory process can never go beyond the cause, why interfere with remedial agents at all? Is not the supply precisely equal to the demand? Is not the one the exact expression of the other? Why therefore stimulate any more than restrain? Why not fear increased action from stimulants as much as decreased action from blood-letting and other antiphlogistics? Is there not danger of disturbing the harmonious relation which exists between cause and effect?

In reply to this it is claimed by Dr. Todd, that to promote these conservative reactions vital power must be upheld by stimulants; but where vital power is feeble, disintegration, it will be remembered, is correspondingly feeble, and increased determination of blood to the part is not therefore demanded; indeed it would impose a burden—the determination of blood already existing being but an expression of the cause which necessitates it. Why not rather by some means seek to arrest disintegration, which is assumed to be the *primary condition*, and thereby lessen the demand for increased flow of blood to the part?

These views, it will be seen, are not in harmony with the admirable teachings of Paget in his Surgical Pathology. It is true, he locates the prime cause of inflammation in "the various perversions of the necessary conditions of healthy nutrition." These sometimes have their origin in the blood-vessels, sometimes in the blood itself, sometimes in the nervous endowment of the part, and sometimes in the condition of the part to be nourished. Inflammation may be irritated by the perversion of any one of these conditions of nutrition; and let the prime link in the chain of morbid action be what it may, impairment or suspension of the nutrition of the inflamed part is the invariable consequence. Indeed, the impairment of formative capacity and tendency to degeneration and death of the part, is that which most plainly distinguishes inflammation from healthy hyperæmia. In one case we have increased flow of blood, with corresponding activity of the nutrition of the part; in the other there is increased flow of blood, with partial obstruction and degeneration of the part; in one instance the hyperæmia imposes a burden; in the other it supplies a want. The condition of one gradually and imperceptibly passes into the other; and it is as difficult to point out the exact boundary line which separates *active congestion* from inflammation, without the aid of high magnifying powers, as it would be to tell where one shade of the rainbow begins and the other ends. And yet the practical distinction must be recognized, for it lies at the foundation of all rational views of the treatment of inflammation. In the early stages of inflammation we may be able, by remedial agents, to restore one or more of the disturbed conditions of normal nutrition, and thereby avoid the invariable sequence—a lowered vitality, with a tendency to degeneration and death of the part. But if we start with the general proposition that there is no destruction in the types of inflammation, that vascular excitement is in every stage conservative and salutary in its tendency, and seek to aid it by direct stimulation, we cannot but regard the consequences as often disastrous in the extreme.

That Dr. Todd's Lectures, however, are full of most valuable practical illustrations, no one can for a moment deny. His views as to the nature and treatment of rheumatic fever are peculiarly able and suggestive. He reviews, at length, the various plans of treatment which have been suggested and adopted by their respective advocates, and, for convenience of arrangement, adopts the following order: The first plan is that by venesection; 2, moderate bleeding and diaphoretics; 3, mercury; 4, colchicum and guaiacum; 5, opium; 6, quinine; 7, *treatment by elimination.* The latter plan he regards as the most rational and successful. With Fuller, Williams, Watson, and others, he thinks it probable that the *materies morbi* in rheumatic fever is *lactic acid*, or some analogous agent, and the most natural emunctories of this are the skin and kidneys, a certain portion of it, perhaps, being carried off through the alimentary canal. His indications of treatment are, then, to promote the action of the skin, the kidneys, and the bowels; to use antacid remedies; and to give large quantities of fluid for the free dilution of the *materies morbi.* For the purpose of promoting the action of the skin, he is partial to the use of opium, especially if combined with nitre and ipecacuanha; and he thinks

"The best alkali, on the whole, is the bicarbonate of potassa, which may be given in large and often-repeated doses—a scruple or half a drachm every third hour. Sometimes the acetate of potassa answers very well in similar doses, and many physicians prefer it to any other alkaline salt."

And as a purgative he uses alkalines—the "white mixture" of the hospital, containing magnesia and sulphate of magnesia. The local treatment is also thoroughly discussed. The practice of leeching he regards as not only useless, but often positively hurtful—"You render the disease more erratic, and less amenable to subsequent treatment." When the joints are swollen and painful, great ease may be obtained by enveloping them in a large quantity of soft carded cotton—commonly called *cotton-wool*—over which wrap a sheet of oiled silk, so as to keep the joint in a complete vapor-bath. "It affords great relief, supports and keeps the limb steady, and at the same time promotes sweating." In case, however, the swollen and painful state of the joints does not yield to the cotton wool and oiled silk, he adds:—

"I have no hesitation in advising you to apply blisters. * * * I do not think it advisable to apply *large* blisters; on the contrary, they are injurious, and their use is to be deprecated. * * * * You must not let the size exceed that of a crown-piece. It is better to apply two or three small blisters in rapid succession, and to different parts of the joint, than one large blister."

Our author is somewhat peculiar in his advocacy of the application of blisters in the early stages of rheumatic inflammation :—

"I believe the dread which some physicians had, and have, of applying blisters near inflamed parts—as near an inflamed lung, or pleura, or pericardium—is due to their having used blisters of too great a size. I have applied them very early to rheumatic joints in numerous cases, and always with more or less advantage, provided the blisters have not been too large. A very large blister is very apt to do mischief, and augment the inflammation of the joint; but a small one, varying in size from that of a crown to a half-crown, is almost invariably beneficial. When a very copious effusion has taken place into a joint, the plan of applying two or three small blisters in succession, at different parts of the joint, provided the first should fail in getting rid of the effusion, is productive of the best effects."

And, in the closing of his last lecture on this subject, he calls attention to a sign which, he says, has often led him to pursue an altered course of treatment :—

"When the patient has begun to pass pale urine, in good quantity, either without precipitate, or with a greater or less quantity of pale lithates, you will almost invariably find that he will be the better for a more generous treatment, even although the articular affection still continue troublesome. You may give him ammonia, or quinine and sulphuric acid, and in many instances you may give wine or brandy; and I have been astonished at the rapidity of the progress of cases under this altered treatment; patients, whose symptoms had been stationary for two or three days, have, under the circumstances and treatment I have described, become convalescent in little more than forty-eight hours."

The lectures on Continued Fever embrace the consideration of the typhoid, typhus, and relapsing forms. Beyond a clear statement of our current literature on these subjects, they contain nothing new. Our author subscribes to the doctrine of Louis, Jenner, Bartlett, and others, of this country, that typhus and typhoid are produced by a distinct, although, doubtless, very similar poison : but he confesses to the difficulty of making out, at all times, a satisfactory diagnosis :—

"Admitting, as I do, the existence among cases of continued fever of two clinical varieties, the typhoid and typhus, I am nevertheless convinced that instances every now and then occur, in which the distinction cannot be made, unless the presence or absence of enteric symptoms alone, or of some other *single* symptom, be taken as diagnostic."

His treatment consists largely of nutrients and stimulants—beef-tea, quinine, carbonate of ammonia, chloric ether, brandy, opium, and blisters. We give the concluding portion of his last lecture on this subject in full:—

"My first rule is this—never give up a fever patient until he is plainly *in articulo mortis*. Patience, perseverance, and steady adherence to a well devised plan of treatment are, in fever cases, often crowned with success under the most discouraging circumstances.

"2. When you undertake a case of this kind, insist upon having a good nurse; give her full instructions, if possible in writing, and require her to keep a note of all food and medicine administered by her. Do not trust to the nursing by relatives.

"3. It is not advisable that you should see your patient too often; once, or at most twice a day, will be, in general, quite sufficient.

"4. Do not be anxious to account or provide for every new symptom or change that may arise. Do not treat symptoms too much, but look to the general condition of your patient. Diarrhœa, delirium, coma, hemorrhage, are the symptoms you should look out for, and be prepared instantly to meet.

"5. Watch the pulse closely, both as to quality and frequency. Always keep in view that increase of quickness is a sure sign of increasing debility, and that the diminution of quickness (when not referable to any cerebral affection) is the reverse.

"6. Never allow a day to pass without carefully examining the abdomen of your patient, and especially the region of the bladder.

"7. Do not be anxious about the action of the bowels, and the so-called secretions. Many a patient in fever has fallen a victim to the *diligentia medici nimia* in improving 'the secretions.' You may make them perfect in color and consistence, and yet your patient will die.

"8. Restrain diarrhœa and hemorrhage, and when, in typhoid fever, you have fairly locked up the bowels, keep them so. Patients will go for four or six days, or even longer, without suffering inconvenience from this state of constipation.

"9. From the first moment of your attendance, let it be your constant and anxious effort to uphold the vital power of your patient, by nitrogenous food given as broths, and carbonaceous food, selected from farinaceous substances, and from alcoholic fluids, such as wine, brandy, or other fermented liquors.

"10. Increasing delirium and coma are signs of increasing debility: both indicate the necessity for additional support; coma is often benefited by freely blistering the nucha and scalp, and even the region of the heart."

In the treatment of erysipelas, Dr. Todd does not attach much importance to the use of the tincture of sesquichloride of iron. His reliance is mainly upon stimulants and nourishing food, administered freely, from the very commencement of the attack. Indeed, he appears to regard alcoholic stimulants as *antidotes* to the erysipelatous poison:—

"If I were to be restricted to any one remedy in the treatment of this disease, I should, assuredly, choose brandy. With a commissariat well supplied with brandy, and simple means to keep the bowels open, I think I could engage to keep erysipelas at a minimum among the wounded in our army in the Crimea."

But it is in the chapters "On the Treatment of Acute Internal Inflammations" that we have the peculiar views of the author most dogmatically enforced. No one can rise from the perusal of these chapters and not award to him at least honesty in most vigorously acting up to his own belief; for on one occasion he remarks: "This patient had altogether about thirty-one pints of brandy, or an average of about a pint a day for a month." And even the delirium which is often associated with acute inflammatory affections, offers no exception to the brandy treatment:—

"This is a fact which I have so often verified, that I am enabled to enunciate it dogmatically, that alcohol, carefully administered from an early period, in small and often-repeated doses, is the best preventive of, and antidote to delirium in acute disease."

Dr. Todd lays much stress upon the *mode of administering* stimulants: "They should be used *early*, with care and watchfulness, in very definite quantities, and not in a vascillating or timid manner." But when thus carefully administered his faith in the use of these remedies may be judged of by the following paragraph:—

"I say it, after mature reflection and a long course of observation, that there is no point of therapeutics so deserving of the study of the earnest-minded physician or surgeon, who is zealous to save life, as that of the action of these agents, both in health and disease."

The lectures "On Pneumonia and its Complications" we regard as among the ablest of the course. The clinical varieties are divided into (1) Simple pneumonia; (2) Pneumonia complicated with *acute gout*, or with *rheumatic fever;* (3) Strumous pneumonia; (4) Typhoid pneumonia; (5) Traumatic pneumonia. These varieties are presented with a clearness and conciseness which are strikingly in contrast with many of our more elaborate publications on this subject. The composition of the urine in pneumonia is fully discussed. It will be remembered that when hepatization of the lung is complete, there is a deficiency of all the *saline* ingredients, and particularly the *chloride of sodium*, (common salt.) Until resolution is established this salt is wholly, or almost wholly, absent from the urine. The presence or absence of this *chloride* may be easily ascertained by a very simple chemical test.

"About a drachm of the urine should be placed in a test-tube and acidulated with a little nitric acid; to this a few drops of the nitrate of silver should be added, when, if any chloride be present, a white flocculent precipitate (*chloride of silver*) will occur; and the *quantity* of chloride in the urine may be roughly estimated by noting the bulk which this precipitate occupies, after having been allowed to subside to the bottom of the test-tube a short time. If, on the other hand, no precipitate takes place on the addition of the solution of nitrate of silver, you may conclude positively and certainly that no chloride is present in the urine."

Two questions in this connection are submitted by the author: 1. What becomes of the chloride of sodium under these circumstances, if it does not pass out in the urine? 2. Why does the chloride of sodium return so quickly into the circulation and urine? Dr. Beale has shown that chloride of sodium accumulates in the *sputa* and in the *inflamed portion of the lung;* there would appear to be some attraction in the inflamed pulmonary tissue for this salt; the precise object of this, however, is as yet a matter of speculation; but it is an interesting question, as suggested by the author, whether a similar state of things occurs in acute inflammations of other organs, or in bronchitis, etc. And he adds:—

"One conclusion may certainly be inferred from these curious chemical details, namely, that the disease of which we are speaking involves profound changes in the chemistry of life—in the interchanges between the blood and the tissues, and in the chemical constitution of the blood itself. And not only so, but the progress of *recovery* from the disease involves analogous chemical changes in an opposite direction, showing that there is in the human system a wonderful power of restoring the injuries inflicted by disease. When we shall have obtained a clearer insight into the recondite processes by which repair is effected in the animal body, we shall be in a better position to assign to our experimental interferences their proper position either as helps or hinderances to this inherent *vis medicatrix.*"

In the treatment of the various forms of pneumonia Dr. Todd makes just discriminations. In the more active and acute forms he uses, as a diaphoretic, tartar emetic and opium; also the liquor ammoniæ citratis or acetatis in large and frequently repeated doses; applying at the same time counter-stimulants over a considerable extent of the surface of the chest, such as mustard, or flannels soaked in warm spirits of turpentine. In typhoid cases, he regards the free use of stimulants as of essential importance, and also regards it as of "immense advantage" to give quinine freely.

The closing lecture, "On the Therapeutical Action of Alcohol," is a remarkable production,—remarkable for the boldness of the author's views in the use of alcoholic stimulants, even in acute inflammations.

"Alcohol," he says, "in some form or other, is a remedy whose value can scarcely, I think, be over-estimated, and one upon which, when carefully administered, I rely with the utmost confidence in a great number of cases of disease which are at all amenable to treatment."

Again he adds:—

"Alcohol may be employed in all those diseases in which a *tendency* to depression of the vital powers exists; and there are no acute diseases in which this lowering tendency is not present."

It will be seen from the following paragraph that he discards the popular notion that alcohol causes inflammation or congestion :—

"I apprehend that in all cases of brain affection from alcohol, the symptoms are due not to congestion nor to inflammation, but to a simple poisoning of the nerve fibre and nerve cell, by the spirits. It is the same with chloroform and sulphuric ether when inhaled. They act directly on the nerve fibre and nerve cell, and not by producing congestion. If you soak a living nerve in chloroform for a certain time, it will refuse to propagate the nervous force under chemical, mechanical, or electrical stimuli."

We have thus brought to a close our review of the last of the lamented author's productions. For his learning and ability we have ever entertained the most profound respect; few men, living or dead, have shed greater lustre upon our profession; and none certainly worked with a more ardent desire to arrive at truth. But the very brilliancy of his genius and the lustre of his name lead us to regret the more the *ultimatum* to which he was fast arriving, namely, that all diseases consist in depression of the vital forces, and that brandy is the remedy. In his life he was justly regarded as one of the great lights of the profession: in his death we fear he has bequeathed to us, in his "Clinical Lectures on Certain Acute Diseases," a doubtful legacy.

